# Microgrooved-surface topography enhances cellular division and proliferation of mouse bone marrow-derived mesenchymal stem cells

**DOI:** 10.1371/journal.pone.0182128

**Published:** 2017-08-28

**Authors:** Jitendra Kumar Chaudhary, Pramod C. Rath

**Affiliations:** Molecular Biology Laboratory, School of Life Sciences, Jawaharlal Nehru University, New Delhi, India; Instituto Butantan, BRAZIL

## Abstract

Mesenchymal stem cells’ (MSCs) fate is largely determined by the various topographical features and a range of extracellular matrix (ECM) components present in their niches. Apart from maintaining structural stability, they regulate cell morphology, division, proliferation, migration and differentiation among others. Traditional MSC cultures, which are mainly based on two-dimensional smooth surfaces of culture dishes and plates, do not provide topographical cues similar to *in vivo* three-dimensional niches, impacting various cellular processes. Therefore, we culture the mouse bone marrow-derived MSCs on microgrooved bearing surface, partially mimicking *in vivo* reticulated niche, to study its effect on morphology, pluripotency factor-associated stemness, cell division and rate of proliferation. Following culture, morphological features, and MSC-specific marker gene expression, such as CD29, CD44, Sca-1 along with HSC (Haematopoietic stem cell)-specific markers like CD34, CD45, CD11b were evaluated by microscopy and immunophenotyping, respectively. HSC is another type of bone marrow stem cell population, which concertedly interacts with MSC during various functions, including haematopoiesis. In addition, mesenchymal stem cells were further analyzed for gene expression of pluripotency-associated transcription factors such as Oct3/4, Sox-2, Nanog and Myc, as well as differentiated into adipocytes, osteocytes and chondrocytes. Our results show that microgrooved surface-cultured mesenchymal stem cells (MMSCs) expressed higher levels of expected cell surface and pluripotency-associated markers and proliferated more rapidly (2–3×fold) with higher percentage of cells in S/G2-M-phase, consequently giving rise to higher cell yield compared to standard culture flask-grown cells (MSCs), taken as control. Furthermore, both MSCs and MMSCs showed considerable accumulation of intracellular lipid-droplets, higher alkaline phosphatase activity and secretion of extracellular matrix that are characteristics of adipogenesis, osteogenesis and chondrogenesis, respectively.

## 1. Introduction

Mesenchymal stem cells (MSCs), also called as multipotent mesenchymal stromal cells, have been isolated from bone marrow, adipose tissue, placenta, and cord blood of human, mouse, rat, porcine, rabbit, dog and equine amongst other species [1–6]. They show differential morphology, growth rate, proliferation and differentiation potential, transcriptomic/proteomic signature depending on their source of origin and biophysical cues such as cell culture media, fetal bovine serum, growth factors, as well as surface topography and kinds of extracellular matrix used during the culture. MSCs, isolated from bone marrow, show a range of cell surface markers such as CD29, CD44, Sca-1 which are used for their characterization and isolation [7–9]. Under optimal conditions and cocktail of differentiation-inducing factors, they could be differentiated into orthodox mesodermal cells like adipocytes, osteocytes, chondrocytes and functional ectodermal cells like neurons, glial cells, and hepatic cells, an endodermal cell lineage [10–13]. Owing to these intrinsic properties, MSCs are being investigated worldwide for cell and tissue therapy, both *in vitro* and in animal models so as to make them therapeutically useful for various tissue- and neuro-degenerative diseases like osteogenesis imperfecta [14], rheumatoid arthritis [15], diabetes [16], acute graft-versus-host diseases [17], infarcted myocardium [18], Alzheimer’s Disease [19] and Parkinson’s Disease [20] amongst others. Therefore, taking above prospects into consideration, we aim to develop deeper insights into method of isolation and culture so as to obtain pure and high yield of MSCs suitable for downstream experimentation and various therapeutic purposes. Originally, A. J. Friedenstein and his colleagues pioneered MSC culture by virtue of intrinsic physical property of mesenchymal stem cells that help them get adhered on the surface of plastic dish/flask [6, 21]. In pursuit of improvement to existing conventional methods, including the original one, a number of techniques and modifications have been developed, such as seeding cells at different cell density, on surfaces with three dimensional topographical features, using different culture media along with varying concentrations of fetal bovine serum and even serum-free medium, [7, 9, 22–24], cell surface-based negative [25] and positive [26] selections, cell sorting, application of conditional/specialized media [27], and so forth.

The interaction of MSCs with extracellular matrix plays an important role in niche formation and MSC functions, as well as working of other bone marrow cells like haematopoietic stem cells (HSCs) [6]. For instances, MSCs seeded on extracellular matrix (ECM) like laminin, collagen and human fibroblast-derived extracellular matrix (hECM)- modified surfaces show enhanced cellular proliferation with higher S-phase percentage cell population [24, 28–31]. Similarly, phage-based supramacromolecular 2D assembled films have been used to study the film’s topographical features on the proliferation and differentiation of MSCs. Such phage-based topographical fabrication has been found to be quite compatible for culturing MSCs, and also induces osteogenic differentiation with highly vascularized bone regeneration [32]. Despite several advantages, abovementioned methods have their own limitations. For examples, many growth factors and ECM are known to be capable of untowardly changing/modifying various cellular processes and hence consequent pleiotropic effects, interfering with crucial results and interpretation [33]. In addition, these methods may have several other limitations such as cell yield and prohibitive cost factor.

The stem cell properties of MSCs include self-renewal, proliferation, pluripotency and multilineage differentiation. Pluripotency is achieved and maintained by functional regulatory network that promotes expression of pluripotency genes, such as Oct3/4, Nanog, Myc, Sox-2, and suppresses expression of differentiation-associated genes. Many studies report that MSCs show dynamic expression of Oct3/4, Nanog. Overexpression of Oct3/4 and Nanog in MSCs enhances rate of cellular proliferation and differentiation potential, while knockdown inhibits the same, indicating their importance in maintenance of MSCs’ properties, including self-renewal, stemness and proliferation [34–35].

Considering aforementioned empirical evidences and experimental nuances, we present a novel method for culture and expansion of mouse bone marrow-derived mesenchymal stem cells. Our method includes culture of MSCs on artificially created microgrooved-surface, independent of any extracellular matrix or synthetic materials, so as to minimize experimental interference on one hand, and considerably reduce time and prohibitive cost on the other. Following culture, we evaluated the purity of cells by immunostaining/immunophenotyping and FACS analysis, stemness by checking expression of pluripotency-associated transcription factors such as Oct3/4, Sox-2, Nanog and Myc, and multipotency by tri-lineage differentiation into cells of the mesodermal lineage, i.e., adipocytes, osteocytes and chondrocytes. Flow cytometric analysis was carried out to analyze the role of microgrooved topography on cell cycle and consquent increase in rate of cell proliferation.

## 2. Materials and methods

### 2.1 Reagents, culture media, antibodies

Dulbecco’s Modified Eagle Medium-High Glucose (DMEM-HG: 4500 mg/500 ml) (cat. no. D 5796), Dulbecco’s Modified Eagle Medium-Low Glucose (DMEM-LG: 1000 mg/500 ml) (cat. no. D 6046), Fetal Bovine Serum heat-inactivated (FBS; cat. no. F4135), Penicillin/Streptomycin/Amphotericin B solution (cat. no. A5955), 0.25% Trypsin/EDTA solution (cat. no. T4049), Dexamethasone (Dex.; cat. no. D2915), Isobutylmethylxanthine (IBMX; cat. no. I 7018), Insulin solution (10 mg/ml; cat. no. I 9278), Indomethacin (cat. no. I7378), Linoleic Acid (cat. no. L5900), Sodium Phosphate dibasic (Na_2_HPO_4_, cat. no. S7907), Sodium Phosphate monobasic (NaH_2_PO_4_; cat. no. S8282), Sodium Selenite (cat. no. S5261), Transferrin (cat. no. T8158), Albumin Bovine Serum (cat. no. A9647), L-Ascorbic acid-2-phosphate sesquimagnesium salt (cat. no. A8960), Weigert’s Iron Hematoxylin solution (cat. no. HT1079), Safranin O (cat. no. 84120), Oil red-O (cat. no. O0625), Trypan blue (cat. no. T8154), Alkaline Phosphatase detection kit (cat. no. Kit 86 R), β-Mercaptoethanol (cat. no. M7522), Retinoic acid (cat. no. R2625), TRI Reagent (cat. no. T9424), Diethyl pyrocarbonate (cat. no. D5758), Poly-L-lysine hydrobromide (cat. no. P2636), Ethidium Bromide (cat. no. E8751), and Agarose (cat. no. A9539) were purchased from Sigma-Aldrich, USA. TGF-β_3_ (Transforming Growth Factor- β_3_; cat. no. 100–36) was from Peprotech, USA. Serological pipettes, 5 ml and 10 ml, sterile and individually wrapped (cat. no. 4051 and 4101, respectively), 15 ml and 50 ml polypropylene centrifuge tubes, sterile (cat. no. 430052 and 430291, respectively), 25-cm^2^ cell culture flask, polystyrene (cat. no. 430639), six-well cell culture plate (cat. no. 3506), 0.20 μm low protein binding sterilization syringe filter, Poly Ether Sulfonate (cat. no. 431229) were from Corning, USA. Cell strainer, 70 μm, nylon (cat. no. 352350), and FITC-labelled: anti-CD44 (cat. no. 553133), anti-CD45 (cat. no. 553079), Rat IgG2b k Isotype control (cat. no. 553988) antibodies, Sheath fluid (cat. no. 342003) were from BD Bioscience, and anti-Sca-1 (cat. no. 11–5981), ant-CD34 (cat. no. 11–0341), anti-CD11b (cat. no.11-0112), Rat IgG2a, kappa Isotype control (cat. no. 11–4321), and PE-labelled anti-CD29 (cat. no. 12–0291), Armenian Hamster IgG isotype control (cat. no. 12–4888) antibodies from eBioscience. The goat anti-rabbit IgG-TRITC labelled antibody (cat no. RTC2) was from Genei. Enzymes and marker/ladder were MMLV-RT (Promega; cat. no. M-1701), Taq DNA polymerase (NEB; cat. no. M0273L), dNTPs (Promega; cat. no. U1330), 100 bp DNA ladder (MBI Fermentas; cat. no. SM1143). Biochemical and molecular biology reagents, such as ethanol, formaldehyde solution (37%-41% w/v), xylene, isopropanol, methanol, glacial acetic acid, giemsa, crystal violet and paraffin wax were purchased from Merck, Germany.

### 2.2. Preparation of microgrooved surface topography

**Fig 1:**
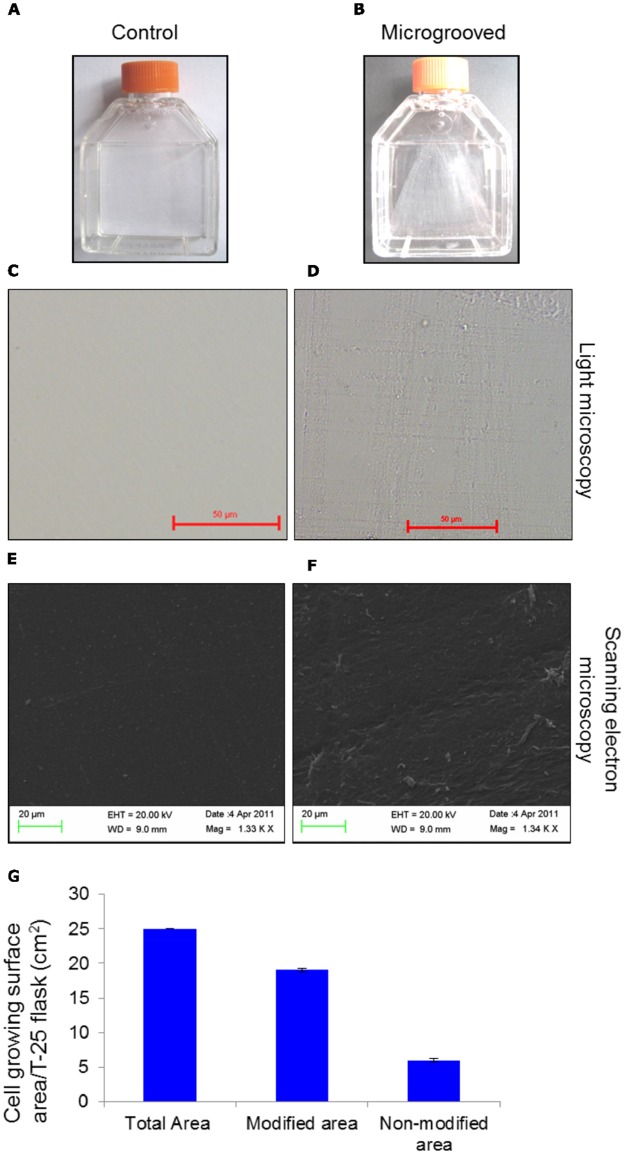
Preparation of microgrooved-topography. (A, B): Bright field images of normal and microgrooved tissue culture flasks, respectively. (C, D): Bright field images of control and microgrooved surfaces, respectively. (E, F): SEM images of control/normal and microgrooved surfaces, respectively. (G): Quantitation of microgrooved/modified surface area (cm^2^) against the total cell growing surface area. Scale bars = 50 μm (C, D), 20 μm (E, F). Results shown are mean ± SEM. ***, P ≤ 0.001.

Microgrooving was carried out on the cell-growing surface of fresh T-25 cm^2^ polystyrene tissue culture flask in laminar flow cabinet. Precisely, after removing cap, cell growing surface was gently scratched by moving tip of steripipette back and forth, and then side to side covering almost entire reachable surface area. The microgrooved surface was washed thrice, each time with 4 to 5-ml phosphate-buffered saline (PBS) with gentle shaking, and then flushing the entire surface over several times with 5-ml steripipette attached to an electric pipette (Fig 1B & 1D). The cell growing surface of standarad culture flask was left unmicrogrooved, and taken as control (Fig 1A & 1C). Thereafter, 2 ml complete medium containing DMEM-HG with 2 mM of L-glutamine, solution of 100 μg/ml streptomycin, 100 unit/ml penicillin, 0.25 μg/ml amphotericin B and 15% FBS were added in control and microgrooved flasks so as to avoid effect, if any, of addition of medium before the cell seeding. The flasks were transferred in CO_2_ incubator for 1–2 hours until cells were ready for seeding. Scanning Electron Microscopy (SEM) of control (Fig 1E) and microgrooved surfaces are shown (Fig 1F) along with measurment of microgrooved area over the total cell growing surface area (Fig 1G).

### 2.3. Isolation and culture of mouse bone marrow-derived mesenchymal stem cells

**Fig 2:**
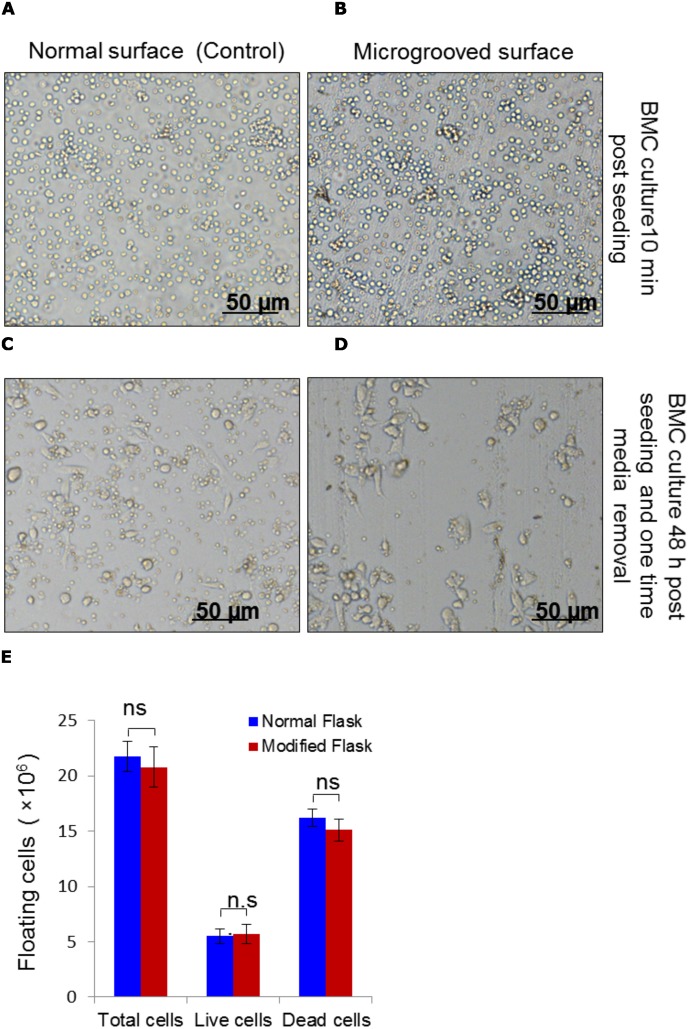
Mouse bone marrow cells (BMCs) culture on normal and microgrooved. (A, B): Bone marrow cells as original source of MSCs were isolated, counted, and then seeded at the density of 1 × 10^6^ cells/cm^2^ in normal and microgrooved flasks, respectively. The images were captured using bright field microscope 5–10 minutes post-seeding. (C, D): The old medium was removed after 48 hours, and pictures of BMC culture were captured. Two types of adhered cells; small spindle-shape and round-shape cells were observed. (E): The floating cells, collected along with old media, were centrifuged and quantitated following 0.4% trypan blue staining to know percentage of attachment of BMCs and dead cells among the floating cell population. Scale bars represent = 50 μm. Results shown are mean ± SEM.

Male C57BL6/J (H2^b^), 4–6 weeks old mice, maintained in on-campus built animal centre facility, Jawaharlal Nehru University (JNU), were used for isolation of bone marrow cells which, upon culture, yielded mesenchymal stem cells. Experiments on mice were approved by Institutional Animal Ethics Committee (IAEC) of JNU, New Delhi, India. Bone marrow cells (BMCs) were isolated using previously published protocol [27]. In brief, each time around 4–5 male mice were euthanized, external surfaces were sterilized with 70% ethanol-soaked cotton swab, and eventually pinned on a customized cork plateform ventral side up. Incisions along the long axis of limbs were made, skins were pulled apart and all the bone-associated muscles were removed. The major limb bones, including femur, humeri and radii were immediately transferred into harvest buffer (Phosphate-buffered saline (PBS) containing 2% fetal bovine serum and 1×antibiotic/antimycotic solution), and then in 35 mm sterile culture dish containing DMEM-HG supplemented with 1× penicillin/streptomycin at RT in culture hood. The epiphyses were cut open with a sterile micro-dissecting scissor, bone marrow was flushed with basal medium consisting of DMEM-HG using 5-ml disposable syringe. Freshly isolated bone marrow was pooled in 15 ml polypropylene tube, dislodged by gentle vortexing, and finally filtered by using 70 μm cell strainer, nylon. A small volume of single cell suspension was mixed with 0.4% trypan blue solution, and then placed on haemocytometer to estimate cell number and cell viability. Thereafter, cells were centrifuged for 5–7 minutes at 400 × g, resuspended in culture medium, and eventually seeded at density 1 × 10^6^ cells/cm^2^ in normal (Fig 2A) and microgrooved (Fig 2B) T 25-cm^2^ (total medium 5 ml/flask) tissue culture flasks. These flasks, with freshely seeded cells, were placed at 37°C, 5% CO_2_ in a humidified incubator. Cells were cultured for 7–10 days with replacement of 4/5 volume of old medum with pre-warm fresh medium, first after 48 hours (Fig 2C & 2D), and subsequently after 3–4 days until cells reached to 60%-70% confluency. These cells are referred as passage 0 MSCs (control flask) and MMSCs (microgrooved flask).

**Fig 3:**
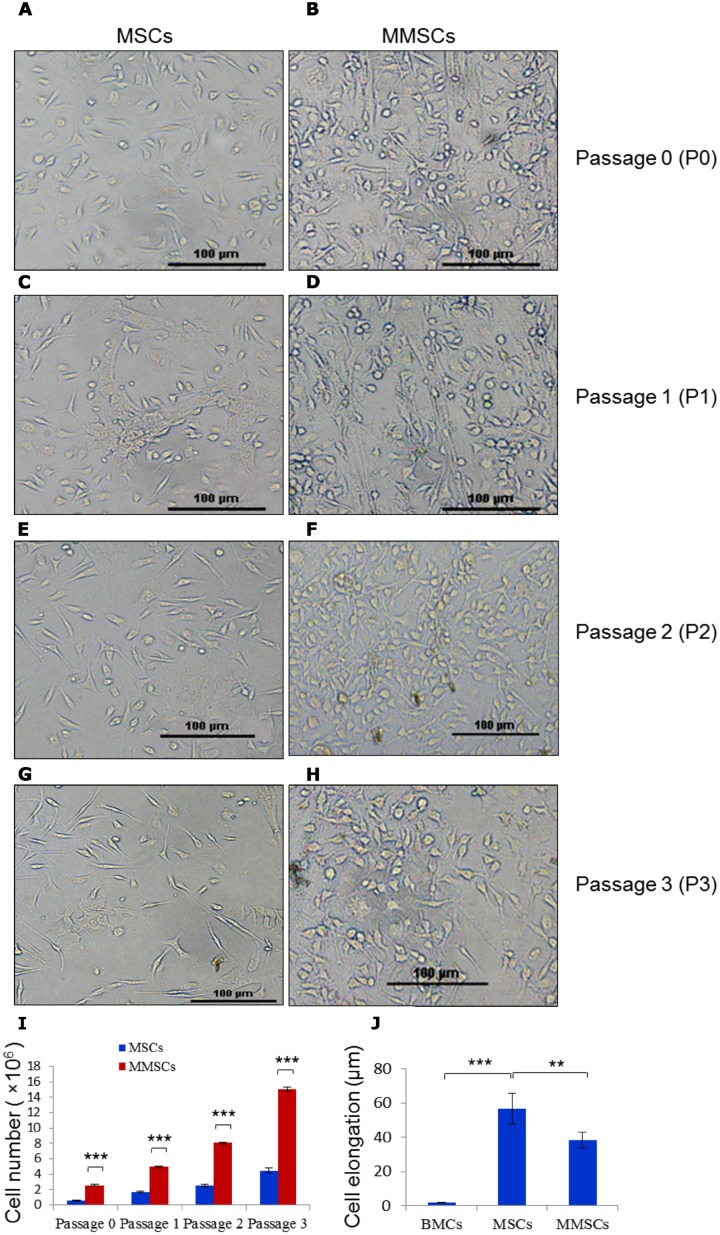
Culture and expansion of mouse mesenchymal stem cells on normal and microgrooved surfaces. (A, C, E, G): Normal surfaces showing MSCs’ morphology and density at P0, P1, P2 and P3, respectively. (B, D, F and H): Microgrooved bearing surfaces showing MMSCs density at P0, P1, P2 and P3, respectively. (I): Grouped bar chart showing quantitation of cell yield obtained from normal (MSCs) and microgrooved (MMSCs) flasks at P0, P1, P2, and P3. (J): Bar chart showing measurement of cell elongation of MSCs and MMSCs. Scale bars represent = 100 μm. Results shown are mean ± SEM. *, P ≤ 0.05, **, P≤0.01, ***, P ≤ 0.001.

Once the desired cellular density of culture was reached, cells were washed with 5 ml pre-warm phosphate-buffered saline, and then incubated with 1.5 ml pre-warm (37°C) 0.25% trypsin/1 mM EDTA solution for 1–2 minute at 37°C. Thereafter, trypsin solution was immediately neutralized by adding 2–3 ml complete medium. Passage 0 of mesenchymal stem cells cultured on normal surface (hereafter refereed to as MSCs) and mesenchymal stem cells cultured on microgrooved surface (hereafter refereed to as MMSCs) were seperately collected, centrifuged for 5 minutes at 400 × g, and reseeded at 0.6–0.7 × 10^6^ cells/5ml/25 cm^2^ in T-25 cm^2^ flasks. These cells were further cultured to obtain passage 1 (Fig 3C & 3D). Similarly, passage 2 (Fig 3E & 3F), and passage 3 (Fig 3G & 3H) were obtained by culturing and plating the cells. Passage 3 MSCs and MMSCs were used for all experiments unless specified otherwise. The MSCs’ and MMSCs’ yields at each passage were calculated and plotted as bar chart (Fig 3I). Furthermore, cell elongation is measured using ImageJ software, and data were plotted by comparing with the measurments of bone marrow cells (Fig 3J).

### 2.4. Scanning electron microscopy of MSCs and MMSCs

**Fig 4:**
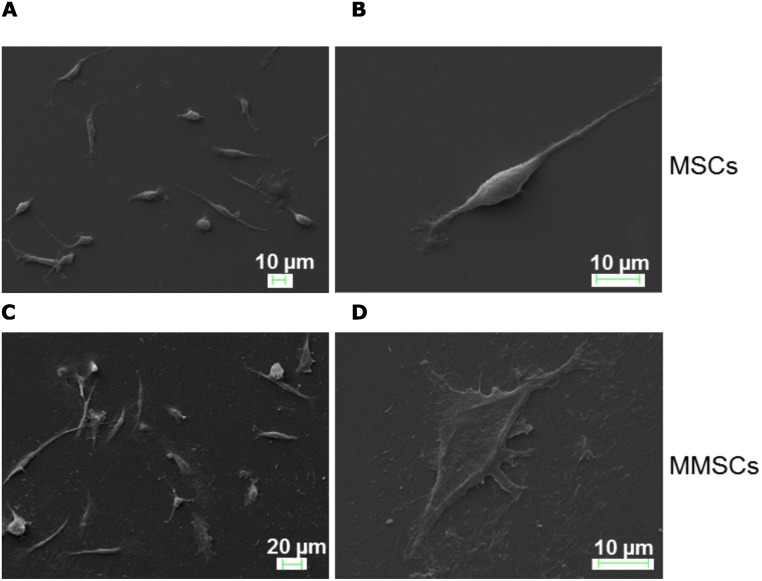
Study of morphological heterogeneity and morphology of P3 MSCs and MMSCs. (A): SEM image depicting three distinct cell populations in the P3 MSCs culture: spindle-shaped, cuboidal or flattened and extremely small rapidly proliferating cells (RS cells). (B): SEM micrograph of a single P3 MSC showing typical spindle-shape morphology with oval-shape nucleus in the centre of the cell. (C): SEM image of P3 MMSCs’ culture cells showing spindle-, flattened- and round-shaped morphologies. (D): SEM micrograph of a typical spindle-shaped P3 MMSC. Scale bars = 10 μm (A, B, D), 20 μm (C).

Once confluent, passage 3 MSCs and MMSCs were washed with 1× PBS, and fixed in 2.5% glutaraldehyde freshly constituted in 0.1 M sodium phosphate buffer (pH 7.2) overnight at 4°C. On the next morning, fixative was carefully drained off, cells were washed with 1× PBS, and thereafter, dehydrated using gradual concentrations of ethanol (30%, 50%, 70%, 90% and 99.9%) for 15 to 20 minutes in each, respectively. Dehydrated cells were sputter coated with gold in the sputtering machine and images were captured using scanning electron microscope (Zeiss EVO40) (Fig 4A & 4B (MSCs); 4C & 4D (MMSCs). Procedure was followed as per published report [36].

### 2.5. Immunostaining and immunophenotyping of MSCs and MMSCs

**Fig 5:**
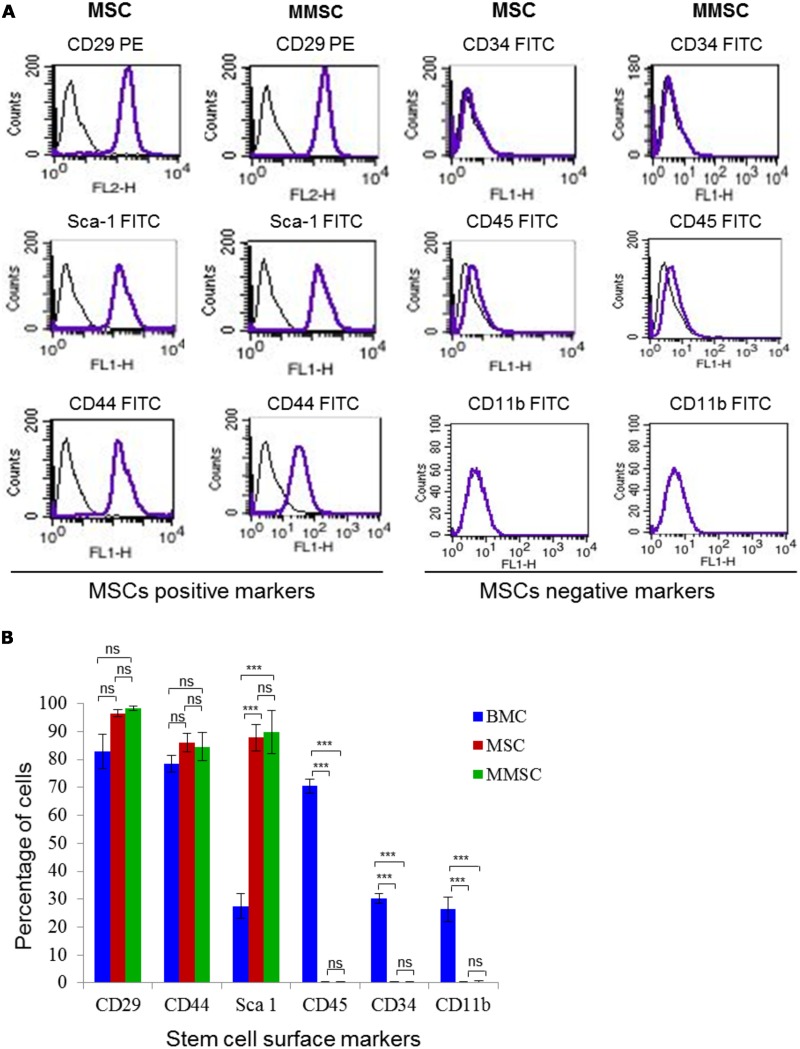
Immunophenotypic characterization of P3 MSCs and MMSCs. (A): Immunophenotyping of the MSCs and MMSCs with respect to well-established mesenchymal-lineage markers such as CD29, CD44, Sca-1, CD45, CD34 and CD11b. The P3 MSCs and MMSCs were stained with PE-conjugated anti-mouse CD29 and FITC-conjugated anti-mouse CD44, Sca-1, CD34, CD45 and CD11b antibodies. The expression of each marker is shown along with the respective isotype control. (B): Grouped bar chart showing quantitation of cell surface markers on P3 MSCs and MMSCs along with BMCs, and data was expressed as mean ± SEM. Abbreviations: PE, phycoerythrin; FITC, fluorescein isothiocyanate.

Both MSCs and MMSCs were harvested separately at the end of passage 3 following trypsinization. Cells were collected and washed with PBS, centrifuged at 400 × g, 5–6 minutes at 4°C, and then resuspended in ice-cold PBS containing 2% FBS. Cell density was adjusted at 1 × 10^6^ cells/50 μl of 2% FBS in ice cold-PBS, aliquoted and stained seperately in dark with PE-conjugated anti-mouse CD29 or FITC-conjugated anti-mouse CD44, Sca-1, CD11b, CD34, CD45 along with their respective isotype control antibodies. Besides, unstained control cell aliquotes were kept separately. Following antibody incubation for 30–40 minutes at 4°C in dark, unbound antibodies were removed by washing cells twice with 1 × PBS followed by centrifugation for 6–7 minutes at 350 × g, 4°C. Cell viability was assessed by adding propidium iodide staining solution at 0.6 μg/10^6^ cells, 10 minutes before the data was acquired. The concentration of antibodies used is given in Table 1, which was decided as per the published reports [23, 27]. During data acquisition on FACSCalibur (Becton Dickinson, San Jose, CA, USA), nearly 20,000 events were collected from each sample. The data was analyzed with the help of Cell Quest software (Fig 5A), and thereafter quantitation of surface markers was plotted (Fig 5B).

**Table 1:** Panel of antibodies used for immunophenotyping and FACS analyses.

Protein	Flurochrome	Dilution (μg/1X10^6^ cells)	Catalogue no.	Company
CD29	PE	1.0	12–0291	eBioscience
CD34	FITC	1.0	11–0341	eBioscience
CD44	FITC	0.125	553133	BD Bioscience
CD45	FITC	0.25	553079	BD Bioscience
CD11b	FITC	0.5	11–0112	eBioscience
Sca-1	FITC	0.5	11–5981	eBiocsience
Rat IgG2a, k Isotype Control	FITC	0.5	11–4321	eBioscience
Rat IgG2b, k Isotype Control	FITC	0.5	553988	BD Bioscience
Armenian Hamster IgG Isotype	PE	1.0	12–4888	eBioscience

FITC, fluorescein isothiocynate; PE, phycoerythrin.

### 2.6. Tri-lineage differentiation

Trilineage diferentiation of passage 3 MSCs and MMSCs were carried out to assess their differentiation plasticity and potential. The method was similar to previously published protocols with minor modifications [23, 27, 37].

#### 2.6.1. Adipocytic differentiation

Once confluent, passage 3 MSCs and MMSCs were harvested and seeded at density 3000 cells/cm^2^/2-ml complete DMEM medium into a 6-well plate. Thereafter, cells were incubated at 37°C with 5% CO_2_ and allowed to grow and adhere for 48 hours. The growth medium was then removed, and replaced with adipogenic induction medium containing DMEM-LG supplemented with 2% FBS, 1 × antibiotic/antimycotic solution, 1.0 μM dexamethasone, 50 μM indomethacin, 500 nM isobutylmethylxanthine, and 5 μg/ml insulin. MSCs and MMSCs were incubated in humidified CO_2_ incubator with fresh, pre-wram adipogenic medium (2ml/well) provided every third day following removal of old induction medium from each well for 14 days. Details about reagents and solvents used to prepare stock solution are given in Table 2.

**Table 2:** Adipogenic differentiation medium.

Components	Stock conc.	Solvents	Working Conc.
DMEM-LG	100%	-	96.72%
FBS	100%	-	2%
Antibiotic/Antimycotic solution	100 X	-	1X
Insulin solution	10 mg/ml	-	5 μg/ml
Dexamethasone	1.0 mM	Water	1.0 μM
Indomethacin	40 mM	Methanol	50 μM
IBMX	10 mM	Methanol	500 nM

**Fig 6:**
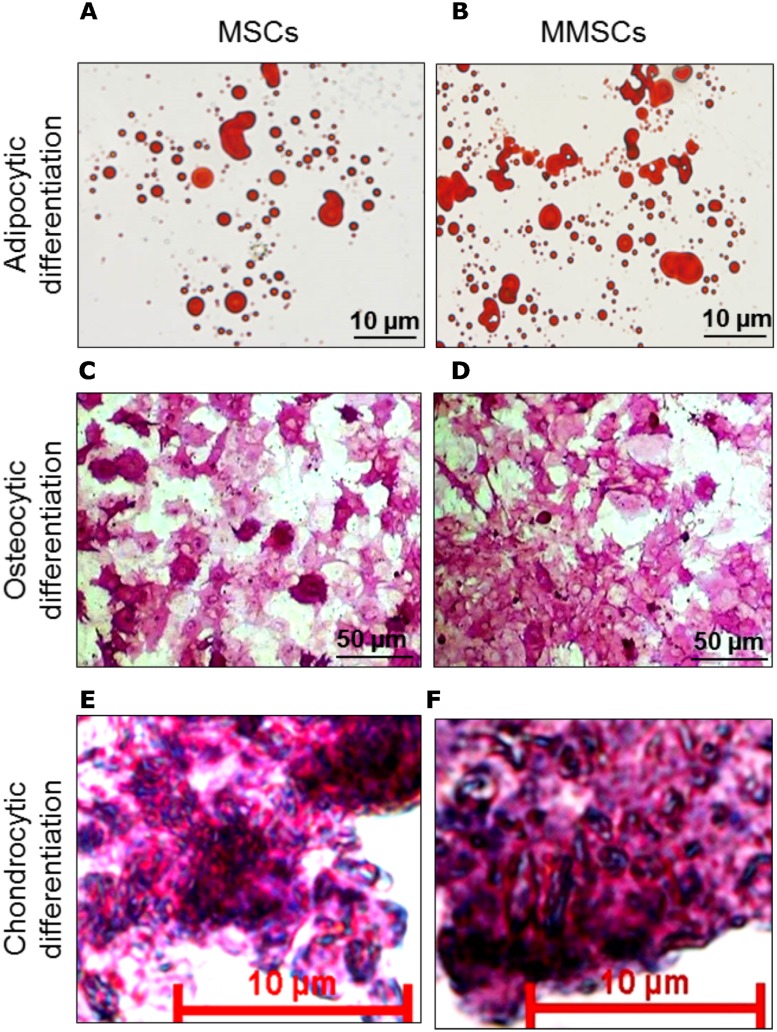
Tri-lineage differentiation of P3 MSCs and MMSCs. (A, B): Oil red O stained adipocytes differentiated from P3 MSCs and MMSCs, respectively. (C, D): Alkaline Phosphatase staining of osteocytes derived from differentiation of MSCs and MMSCs, respectively. (E, F): Chondrocytic differentiation of MSCs and MMSCs followed by Safranin O + haematoxylin staining. Scale bars = 10 μm (A, B, E, F); 50 μm (C, D).

Differentiation was assessed by staining numerous intracellular oil droplets with oil red-O stain following two weeks of adipogenic induction. Oil red-O is a lysochrome (fat-soluble dye) used for staining of cytoplasmic neutral tri-glycerides and lipids. For staining, 1.0 ml/well of 6-well plate of solution containing 3/5 volume of oil red-O (0.5% in isopropanol) and 2/5 volume of water was added at RT for 15–20 minutes. Thereafter, stain was drained off and cells were washed thoroughly with 1× PBS. Images were captured using bright field microscope (Nikon) attached with a camera (DS-Fi1C) (Fig 6A; MSCs & Fig 6B; MMSCs).

#### 2.6.2. Osteocytic differentiation

For osteoblastic differentiation, passage 3 MSCs and MMSCs were seeded at density 3000 cells/cm^2^/2-ml complete medium into a 6-well plate. Thereafter, cells were incubated at 37°C with 5% CO_2_ and allowed to settle and adhere for 48 hours. The growth medium was then removed, and replenished with osteogenic induction medium consisting of DMEM-LG supplemented with 2% FBS, 1× antibiotic/antimycotic solution, 50 μM L- ascorbic acid-2- phosphate sesquimagnesium salt, 10 nM dexamethasone and 10 mM β-glycerophosphate. MSCs and MMSCs were incubated in humidified CO_2_ incubator with fresh, pre-wram osteogenic medium (2 ml/well) that was added every third day for 14 days following removal of old medium. Details about reagents and solvents used to prepare stock solution is given in Table 3.

**Table 3:** Osteogenic induction medium.

Components	Stock Conc.	Solvents	Working Conc.
DMEM-LG	100%	-	94.974%
FBS	100%	-	2%
Antibiotic/Antimycotic	100 X	-	1X
Ascorbic acid	200 mM	Water	50 μM
Dexamethasone	1 mM	Water	10 nM
β-glycerophosphate	0.5 M	Water	10 nM

Osteocytic differentiation was evaluated relying on intracellular alkaline phosphatase (ALP) activity, a marker for osteocytic differentiation. In brief, differentiated cells were washed with 1 × PBS, and then incubated with pre-made staining solution as per manufacturer’s instruction provided with alkaline phosphatase detection kit. Following staining, cells were photographed using inverted bright field microscope (Nikon/ Zeiss) (Fig 6C & 6D).

#### 2.6.3. Chondrocytic differentiation

For chondrogenic differentiation, passage 3 MSCs and MMSCs were harvested separately following trypsinization. Around 0.5–0.6 × 10^6^ MSCs and MMSCs were pelleted seperately in 15 ml polypropylene tubes by centrifuging them at 350 × g for 10 minutes. Thereafter, each cell pellet was kept submerged in 1 ml fresh, pre-warm chondrogenic induction medium consisted of DMEM-HG supplemented with 1 × antibiotic/antimycotic solution, 6.25 μg/ml insulin solution, 6.25 μg/ml transferrin, 6.25 μg/ml sodium selenite, 5.33 μg/ml linoleic acid, 1.25 mg/ml bovine serum albumin, 100 nM dexamethasone, 50 μM ascorbic acid and 10 ng/ml recombinant human TGF-β_3_. Marked tubes with submerged pellet of MSCs and MMSCs were kept upright in humidified CO_2_ incubator with fresh, pre-wram chondrogenic medium (1ml/tube) being provided every third day for 21 days. Details about reagents and solvents used to prepare stock solution is given in Table 4.

**Table 4:** Chondrogenic induction medium.

Components	Stock Conc.	Solvent	Working Conc.
DMEM-HG	100%	-	96.6%
Antibiotic/Antimycotic	100 X	-	1 X
Insulin solution	10 mg/ml	-	6.25 μg/ml
Transferrin human	6.25 mg/ml	Water	6.25 μg/ml
Sodium Selenite	6.25 mg/ml	Water	6.25 μg/ml
Linoleic acid	0.533 mg/ml	Water	5.33μg/ml
Bovine Serum Albumin	125 mg/ml	Water	1.25 mg/ml
Dexamethasone	1 mM	Water	100 nM
TGF-β_3_	0.1 mg/ml	Water	10 ng/ml

Following 21 days of chondrogenic induction, MSCs and MMSCs tubes were taken out, centrifuged at 350 × g. Following centrifugation, supernatant was removed and pellets were now submerged in 1.0 ml freshly prepared 10% neutral-buffered formalin (10% NBF consists of 0.35 g Na_2_HPO_4_, 0.35 g NaH_2_PO_4_, 10 ml of 37% formaldehyde solution and 90 ml double distilled water) for 24 hours at RT. Fixed pellets were carefully removed with the help of soft brush and transferred to 70% ethanol for 30 minutes, and then 90% and 99.9% ethanol for 15 minutes, with two changes in each. Once dehydrated, pellets were transferred in xylene for 15 minutes, and then in a mixture of equal volume of molten wax and xylene for 5 minutes with three changes, at 60°C, and eventually to 100% molten wax for 10 minutes. After xylene-wax processing, blocks were made by pouring molten wax initially up to nearly 50% height of L-shaped metallic frame and then placing processed MSCs and MMSCs-derived chondrocytic pellets right in the centre and filling the remaining space with molten wax. This set-up was allowed to solidify overnight. On next day, blocks containing MSCs and MMSCs pelletes were trimmed peripherally with sharp razor and mounted on block holder of rotatory microtome. Sections of around 5 μm thickness were cut on rotary microtome and then transferred on egg albumin-coated slides. Tissue sections were made adherered by warming slides on slide warming table for 10–20 minutes at pre-set temperature of 40°C. Tissue sections were deparaffinized by immersing them in xylene solution for 10 minutes, and then redehydrated in a series of downgrade ethanol beginning with 99.9%, 90%, 70%, 50% and finally 30% for 15 minutes in each. Sections were stained with Weigert’s Iron Hematoxylin Solution set for 2 to 3 minutes and then carefully washed with tap water for 5 minutes. Furthermore, sections were stained with another staining solution, 0.1% safranin O, for 5 minutes, dehydrated twice in 95% and 99.9% ethanol for 2 minutes each and eventually in xylene twice, each for 5 minutes. Finally, double-stained (hematoxylin-safranin O) sections were mounted in diphenylxylene (DPX) and pictures were captured under optical microscope. Safranin O stain and Weigert’s Iron Hematoxylin solution stained extracellular proteoglycans red and nuclei blue/black, respectively (Fig 6E & 6F).

### 2.7. RNA extraction and RT-PCR analysis

Once passage 3 cells became confluent, total RNAs were extracted and purified from 1–2 × 10^6^ BMCs, MSCs and MMSCs by, using Trizol reagent and employing the method provided with the kit. Briefly, P3 MSCs and MMSCs were harvested, and immediately fixed in TRI Reagent^r^ at -80°C. Following extraction and purification, RNAs were quantified using Nanodrop 2000c spectrophotometer (Nanodrop), and their integrity was assessed on 1% agarose gel containing ethidium bromide stain. Thereafter, cDNA was synthesized using reverse transcriptase enzyme (RT) in 25 μl reaction mixture as per the recommended protocol. The control tube did not contain reverse transcriptase enzyme.

**Fig 7:**
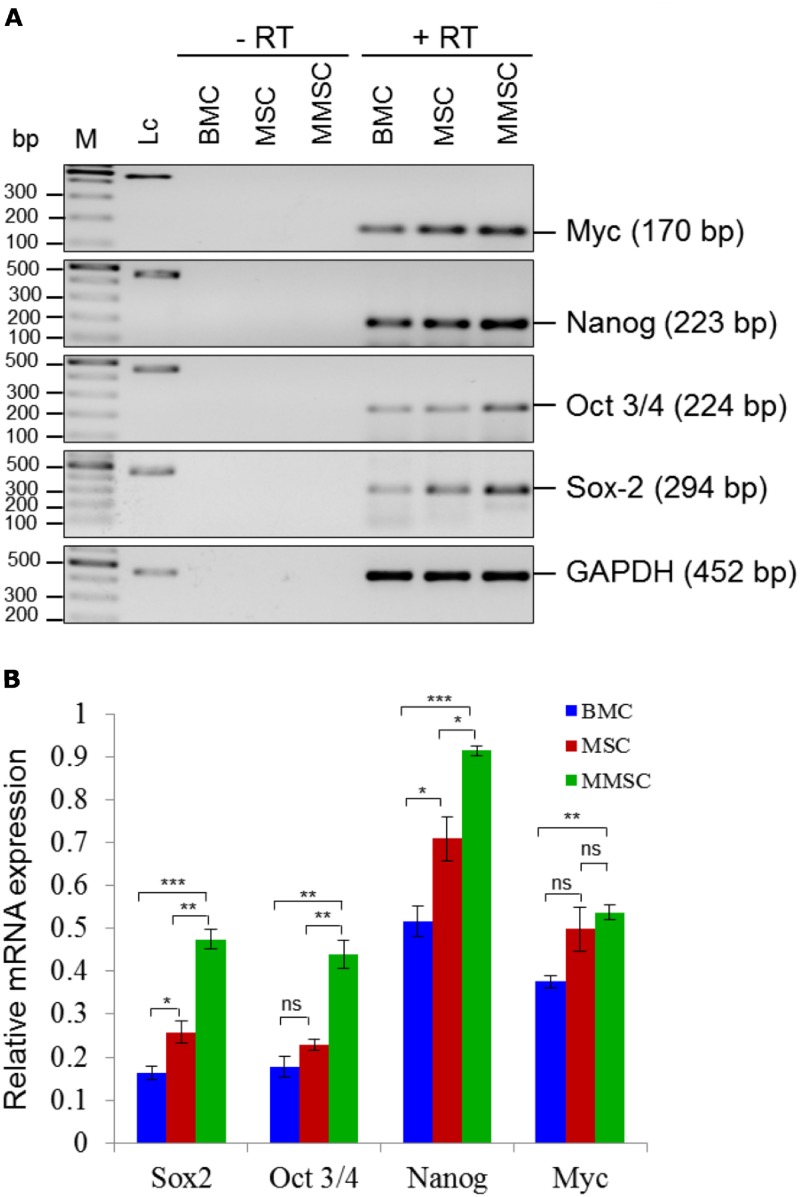
Expression of pluripotency-associated transcription factors in BMCs, MSCs and MMSCs. (A): The basal expression of pluripotency-associated markers like Oct3/4, Sox-2, Nanog and Myc was measured at RNA level by RT-PCR and found to be considerably high in MSCs compared to the BMCs, and this relatively higher basal expression was even significantly higher in MMSCs. (B): Grouped bar diagram showing quantitation of pluripotency markers. Results shown are mean ± SEM of three independent experiments. *, P ≤ 0.05, **, P≤0.01, ***, P ≤ 0.001, P ≥ 0.05, n.s., not significant. Abbreviations: bp, base pair; M, 100 bp DNA ladder used as marker; Lc, loading control (452 bp); RT, reverse transcription.

Pluripotency-associated genes are expressed in all kinds of adult stem cells. Therefore, cDNAs for genes encoding Oct3/4, Sox-2, Nanog and Myc were amplified using Taq polymerase on PCR thermal cycler, considering GAPDH as an internal control from BMCs, MSCs and MMSCs. PCR products were quantified and normalized using GAPDH and a composite bar graph was plotted (Fig 7A & 7B). Primer sequences and their respective annealing temperatures used for the cDNA amplification by RT-PCR are given in Table 5.

**Table 5:** Primers used for RT-PCR.

Gene	Forward primer sequence	Reverse primer sequence	Annealing Temp (°C)
Oct3/4	5’-TCTTTCCACCAGGCCCCCGGCTC-3’	5’-TGCGGGCGGACATGGGGA GATCC-3’	76
Sox-2	5’-TTGCCTTAAACAAGACCACGA AA-3’	5’-AGCTAGACTCCGGGCGATGA-3’	62
Nanog	5’-CAGGTGTTTGAGGGTAGCTC-3’	5’-CGGTTCATCATGGTACAGTC-3’	57
Myc	5’-AAGTTTGAGGCAGTTAAAATT ATGGCTGAAGC-3’	5’TGACCTAACTCGAGGAGGAGCTGGAATC-3’	83
GAPDH	5’-ACCACAGTCCATGCCATCAC-3’	5’- TCCACCACCCTGTTGCTGTA-3’	60

### 2.8. PI Staining and cell cycle analysis

**Fig 8:**
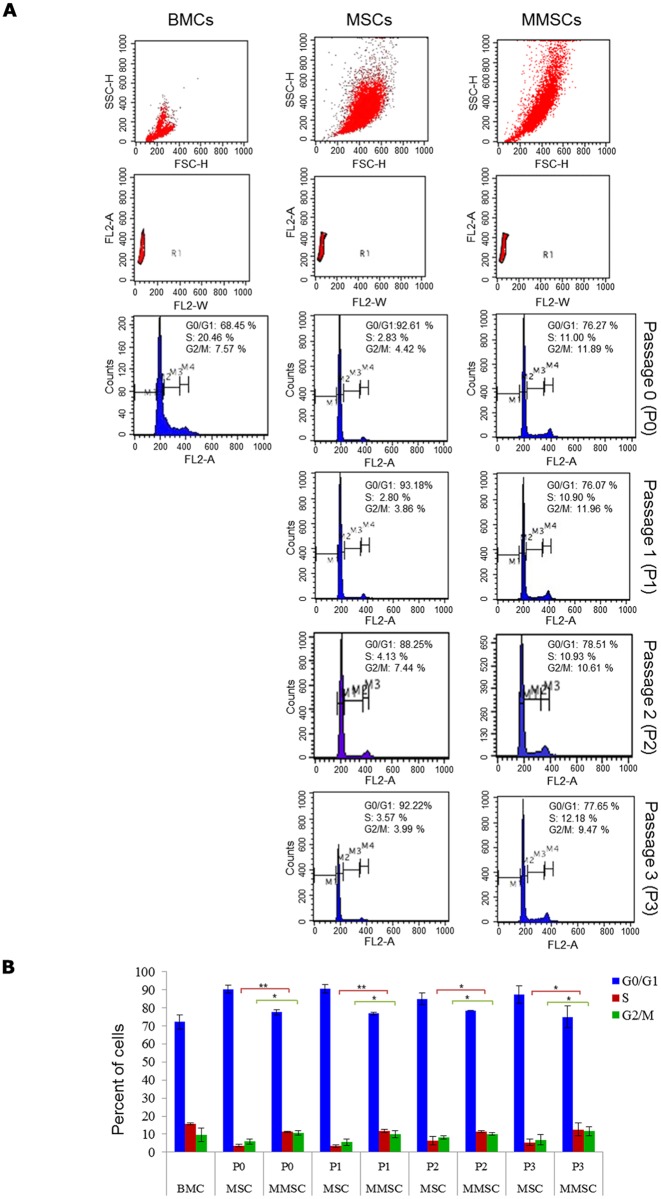
Flow cytometric analysis of cell cycle with propidium iodide. (A): Comparative cell cycle analysis of BMCs, MSCs and MMSCs showing percentage of cell population in various cell cycle stages (G1, S, G2/M). (B): Bar chart showing cell stage-specific quantitation among BMCs, MSCs and MMSCs populations. Abbreviations: FSCs, forward scatter; SSC, side scatter; FL2-A/W, filter 2-area/width. M1, Sub G0/G1; M2, G0/G1; M3, S-phase; M4, G2/M phase. Results shown are mean ± SEM of three independent experiments. *, P ≤ 0.05, **, P≤0.01, ***, P ≤ 0.001, P ≥ 0.05, n.s., not significant.

For cell cycle analysis, freshly isolated BMCs and cultured P0, P1, P2, and P3 MSCs and MMSCs were seperately harvested, washed with cold 1× PBS, and then immediately fixed in 70% ethanol for 2 hours. Thereafter, cells were centrifuged and supernatant/fixative was discarded. Fixed cells were washed twice with cold PBS and incubated with RNase A (200 μg/ml/10^6^ cells) at 37°C for 1 hour. Following RNA digestion, 100 μg propidium iodide/10^6^ cells was added and then incubated on ice for 20 minutes in dark. Finally, data was acquired and analyzed by flow cytometry on FACSCaliber at 488 nm (FL-2) channel (Fig 8A & 8B). The procedure followed is similar to the published reports with subtle modifications customized to suit bone marrow cells and mesenchymal stem cells [38–40].

### 2.9. Statistical analyses

The student’s *t*-test was used to determine significant difference between means of two groups on account of cell yield, cell surface markers and quantitative analysis of gene expression. The difference was considered statistically significant at P<0.05 and data were illustrated as mean ± SEM from three independent experiments.

## 3. Results

### 3.1. Microgrooved surface shows expected cell morphology and surface marker expression

Bright field microscope-acquired images show smooth surface in the standard tissue culture flask (Fig 1A & 1C), taken as control, while microgroove bearing topography shows reticulated pattern all over the cell-growing surface (Fig 1B & 1D). Furthermore, control and microgrooved surfaces were examined at higher magnification by Scanning Electron Microscopy to reveal subtle topographical features (Fig 1E & 1F, respectively). SEM micrographs of microgrooved surface revealed microscopic ridges and furrows covering the entire area that support cell growth during culture. Initially, primary bone marrow cells in culture appeared round-shaped with varying sizes, irrespective of surface topography (Figs 2A & 1B). With passage of time, cells on microgrooved-surface showed relatively more cellular elongation and less number of round cell population compared to control (Fig 2C & 2D). Around 20% cells were found adhered on both normal and microgrooved surfaces by 48 hours of culture (Fig 2E). Later on, MSCs acquired more cellular elongation but less density (Fig 3A, 3C, 3E & 3G) compared to MMSCs (Fig 3B, 3D, 3F & 3H) across all passages (P0, P1, P2 and P3). Likewise, SEM images of passage 3 MSCs (Fig 4A & 4B) and MMSCs (Fig 4C & 4D) displayed typical spindle-shaped morphology, one of the several characteristics of mesenchymal stem cells [4–6].

Immunophenotyping of BMCs, as starting cell population, along with MSCs and MMSCs revealed differential expression pattern of mesenchymal lineage markers such as CD29, CD44, Sca-1, and CD34, CD45, CD11b, hematopoietic stem cell/lineage markers. For instance, BMCs showed positive expression for all markers such as CD29 (82.86 ± 6.29%), CD44 (78.43 ± 3.06%), Sca-1 (27.55 ± 4.50%), CD45 (70.44±2.39%), CD11b (30.18±1.71%) and CD34 (26.36 ± 4.41%). On the contrary, MSCs clearly showed higher positive percentage of expression with respect to CD29 (96.46 ± 1.30%), CD44 (86 ± 3.3%) and Sca-1 (87.83 ± 4.71%), and were almost negative for CD34 (0.39 ± 0.14%), CD45 (0.38 ± 0.08%) and CD11b (0.5 ± 0.19%). Furthermore, compared to BMCs and MSCs, MMSCs showed even higher positive percentage of expression with respect to CD29 (98.33 ± 0.69%), CD44 (84.51 ± 5.09%) and Sca-1 (89.83 ± 7.70%), the markers attributed to bone marrow-derived mesenchymal stem cells, and were almost negative for the haematopoietic markers like CD34 (0.35 ± 0.15%), CD45 (0.39 ± 0.08%) and macrophage marker, CD11b (0.44 ± 0.21%) (Fig 5A & 5B). This reflects similarity of our MMSCs, on account of expression of aforementioned markers, with MSCs reported in published methods [23, 27].

### 3.2. MMSCs show tri-lineage differentiation compared to MSCs

Both MSCs and MMSCs were tested for adipocytic and osteocytic differentiation. This was accomplished by providing them with specific induction media for 2 weeks. After specific period of adipocytic induction, both cells acquired round/oval-shaped morphology with accumulation of numerous round-shaped lipid droplets in their cytoplasm as revealed by oil-red O staining. Likewise, MSCs and MMSCs were checked for alkaline phosphatase activity, a marker expressed in bone cells, following completion of osteocytic induction and differentiation. After differentiation analysis, both MSCs and MMSCs were found to possess equal and comparable potential for adipogenic (Fig 6A & 6B) and osteogenic (Fig 6C & 6D) differentiation.

As for as checking chondrogenic differentiation potential of MSCs and MMSCs is concerned, harvested P3 cells were centrifuged separately in 15 ml polypropylene tubes so as to make them settle down and stick to the bottom of the tubes in the form of pellets. Following three weeks of incubation in chondrogenic inducing medium, MSCs and MMSCs-based pellets increased in size owing to secretion and accumulation of various extracellular matrix components, including collagen II and chondroitin sulflate that were stained by safranin O. Moreover, the pellet following MMSCs (Fig 6F) differentiation showed significant increase in size as well as compactness compared to MSCs (Fig 6E) as revealed following histological analysis.

### 3.3. MMSCs show significantly higher levels of expression of pluripotency-associated marker genes

We also checked the basal and constitutive mRNA expression levels of pluripotency-associated markers such as Oct4, Nanog, Sox2 and Myc in BMCs, MSCs and MMSCs. While MSCs showed insignificant differences in the level of expression of Oct3/4 and Myc, MMSCs showed significantly higher expression of all the aforementioned factors compared to BMCs. Furthermore, except Myc, basal mRNA expression levels of Sox2, Nanog and Oct3/4 were found to be significantly higher in MMSCs compared to MSCs indicating relatively higher pluripotency potential in MMSCs compared to BMCs and MSCs (Fig 7A & 7B).

### 3.4. MMSCs show significantly higer proliferation rate compared to MSCs

To investigate the effect of microgrooved surface on cell cycle, cell division and proliferation, P0, P1, P2, and P3 MSCs and MMSCs were analyzed on account of morphology, cell density and cell cycle profile. The MSCs and MMSCs, seeded at same cell density at the beginning of each passage (P0-P3), showed differential cell density, always with higher density in the microgrooved flask within the same duration compared to control (Fig 3). Furthermore, cell cycle analysis revealed that MMSCs divided rapidly throughout various passages as compared to MSCs. In conformity to above finding, MMSCs showed 3–4× fold higher cell yield as compared to conventional method as seen in MSCs, and this increase can be clearly attributed to enhanced cell cycle, i.e., higher number of cells in S (~3× fold higher) and G2-M phases (~2.5× fold higher) compared to MSCs (Fig 8A & 8B).

## 4. Discussions and conclusions

In this study, we investigated effects of microgrooved-surface topography on cellular proliferation of murine bone marrow-derived mesenchymal stem cells. Topographical features of nano- and micro-scales, fabricated by using various biocampatible synthetic and biological macromolecules, have shown potential effects on various cellular processes, including cell division and proliferation among others [29–31, 36]. In our study, SEM images of microgrooved surfaces revelaed various micro-sized topographical features which were found to have effects on celluar adherence, cell density, morphology, cell division and proliferation. For instance, bone marrow cells, cultured on normal and microgrooved surfaces started showing differential adherence, elongation and morphological pattern within 24–48 hours of cell seeding (Fig 2C & 2D). Furthermore, bright field microscopy of cell cultures showed higher cell density over the microgrooved surface compared to control, across all passages (Fig 3 P0, P1, P2, and P3). Besides being adherant, these cells appeared in different shapes and sizes including spindle-shape, flattened, oval/round shape and even few cells appeared star-shaped. This morphological heterogeneity is consistently maintained through various passages of MSCs and MMSCs cultures with significant variations between them (Fig 3). This cellular feature was found to be consistent with previously published work [41]. Neverthless, the majority of mesenchymal stem cells, towards the later passages, especially P2 and P3 showed typical spindle-shaped morphology in both experimental set-ups (Fig 3), characteristic of mesenchymal stem cells [6, 23, 26–27, 37]. Scanning electron microscopy distinctly revealed the subtle differences in overall morphological appearance, with MSCs being slightly more slender and elongated (Fig 4A & 4B) compared to MMSCs. The flattened and less elongated morphology of MMSCs (Fig 4C & 4D) may be attributed to higer secretion, accumulation and deposition of extracellular matrix (ECM) in cell proximity due to reticulated microgrooved formation, that make the cells tightly cemented and stretched-out over the maximum surface area beneath them, resulting into significantly flattened morphology (Fig 4D) with consequent lower length to width ratio of the cells. Therefore, this lower length to width ratio of MMSCs, compared to MSCs might favour the cellular proliferation as a similar finding has been previously reported in case of human marrow stromal cells [42].

Flow cytometry-based immunophenotyping showed that, compared to MSCs, MMSCs were relatively more positive for mesenchymal stem cell markers like CD29, CD44 and Sca-1, and devoid of haematopoietic stem cell markers like CD34, CD45 and CD11b (Fig 5). This indicates MMSCs being immunophenotypically better and more pure as compared to MSCs obtained following well-established traditional methods [6, 26–27], and that may be used for further experimentation.

Both, MSCs and MMSCs readily differentiated into adipocytes, osteocytes and chondrocytes in their respective induction media (Fig 6). After 14-days of culture in adipogenic differentiation medium, numerous oval/round-shaped cytoplasmic lipid droplets of various sizes appeared, indicating occurrence of the process of adipogenesis (Fig 6A & 6B). Similarly, osteogenic medium resulted in increased cellular alkaline phosphatase (ALP) activity (Fig 6C & 6D), showing osteocytic differentation with similarity between MSCs and MMSCs. Furthermore, alkaline phosphatase positive cells appeared in various sizes and shapes, like star-shaped, rounded and some globular, with differential alkaline phosphatase activity reflected as differential colour intensity, possibly indicating either varying degree of differentiation or osteocytic heterogeneity. On the contrary, histological staining of MMSCs-differentiated pellet showed higer compactness and secretion of several key components of ECM such as collagen II and chondroitin sulflate, resulting into significantly better chondrogenic differentiation compared to MSCs (Fig 6E & 6F). These findings, with regard to differentiation potential of MSCs and MMSCs, are quite comparable with published reports [23, 27, 29, 37].

Previous studies have demonstrated the crucial role of pluripotency genes, such as Oct4 and Nanog in maintaining stemness and self-renewal in embryonic stem cells (ESCs) as well as adult stem cells such as MSCs. Down regulation of Oct4 and Nanog promotes higher expression of developmental and tissue-specific genes, while overexpression in MSCs increases cell proliferation. This finding clearly establishes positive association between level of their expression and cell proliferation. Therefore, MMSCs showing significantly enhanced basal expression of pluripotency-associated markers such as Nanog, Sox2 and Myc and Oct3/4, compared to BMCs and MSCs (Fig 7A & 7B), may be implicated for higher percentage of MMSCs in S/G2-M phases across various passages, and consequently enhanced cellular proliferation as found in our experiment (Fig 8A & 8B), as well as observed in similar conditions by recently published reports [29, 33].

In conclusion, our method resulted into higher yield of pure population of bone marrow-derived mesenchymal stem cells with higher self-renewal, stemness and tri-lineage differentiation potential, and hence, may provide a novel alternative for obtaining relatively better culture and rapid expansion of mesenchymal stem cells, cutting substantially on prohibitive cost and time.

## Supporting information

S1 Table Quantitation of cell yield and measurment of cell elongation. (A). Mesenchymal Stem Cells (MSCs) cultured in standarad flask, starting with the total bone marrow cells and then passage 0 (P0) through passage3 (P3). (B). MSCs cultured in flask with microgrooved surface through various passages (P0-P3). (C). Cellular elongation of both MSCs and MMSCs were measured and compared with bone marrow cells (BMCs).
(TIF)

S2 Table Expression of cell surface markers. Expression of cell surface markers such as CD 29, CD 44, Sca-1, CD 34, CD 45 and CD11b on bone marrow cells (BMCs), mesenchymal stem cells (MSCs) and microgrooved surface-grown mesenchymal stem cells (MMSCs).
(TIF)

S3 Table Expression of pluripotency markers. Expression of pluripotency-associated markers such as Oct3/4, Sox 2, Nanog and Myc in bone marrow cells (BMCs), mesenchymal stem cells (MSCs) and microgrooved surface-grown mesenchymal stem cells (MMSCs).
(TIF)
