# Supplementary figures and images for: Microgrooved-surface topography enhances cellular division and proliferation of mouse bone marrow-derived mesenchymal stem cells
Source: PLoS One. 2017 Aug 28;12(8):e0182128. doi: 10.1371/journal.pone.0182128 (PMC5573154; doi:10.1371/journal.pone.0182128)

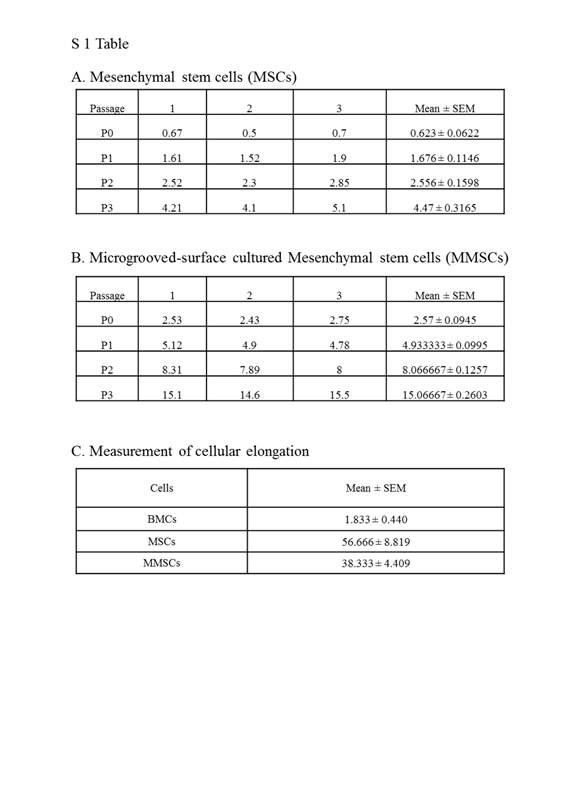

Supplement: S1 Table — (A). Mesenchymal Stem Cells (MSCs) cultured in standarad flask, starting with the total bone marrow cells and then passage 0 (P0) through passage3 (P3). (B). MSCs cultured in flask with microgrooved surface through various passages (P0-P3). (C). Cellular elongation of both MSCs and MMSCs were measured and compared with bone marrow cells (BMCs). (TIF) [file pone.0182128.s001.tif]

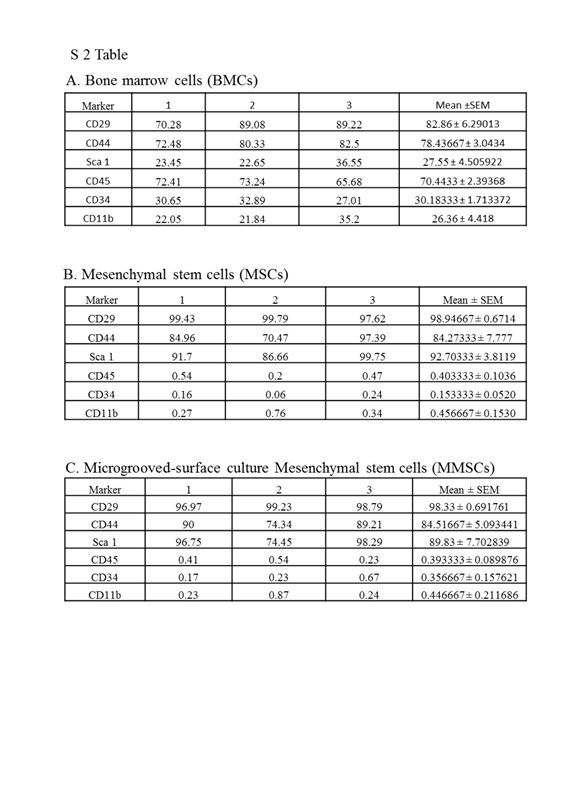

Supplement: S2 Table — Expression of cell surface markers such as CD 29, CD 44, Sca-1, CD 34, CD 45 and CD11b on bone marrow cells (BMCs), mesenchymal stem cells (MSCs) and microgrooved surface-grown mesenchymal stem cells (MMSCs). (TIF) [file pone.0182128.s002.tif]

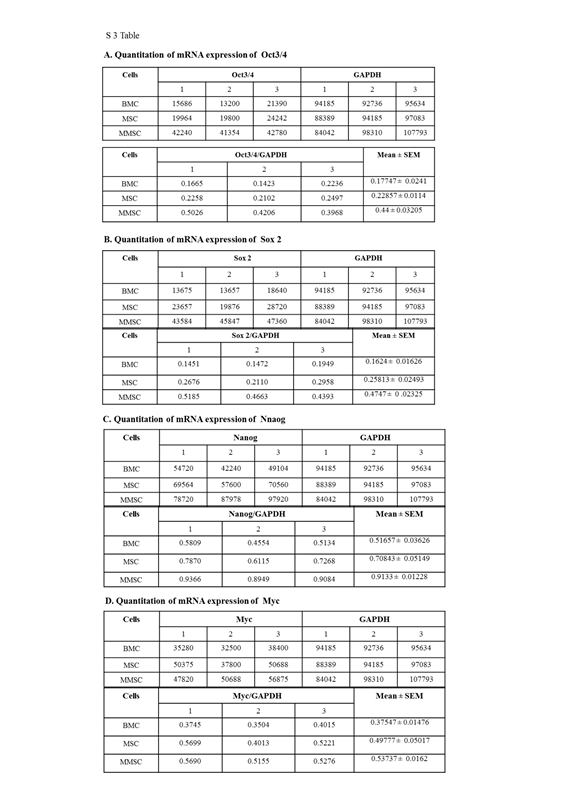

Supplement: S3 Table — Expression of pluripotency-associated markers such as Oct3/4, Sox 2, Nanog and Myc in bone marrow cells (BMCs), mesenchymal stem cells (MSCs) and microgrooved surface-grown mesenchymal stem cells (MMSCs). (TIF) [file pone.0182128.s003.tif]
